# Dietary patterns among Saudis with type 2 diabetes mellitus in Riyadh: A cross-sectional study

**DOI:** 10.1371/journal.pone.0267977

**Published:** 2022-05-05

**Authors:** Abeer Ali Aljahdali, Nahla Mohammed Bawazeer

**Affiliations:** 1 Department of Clinical Nutrition, Faculty of Applied Medical Sciences, King Abdulaziz University, Jeddah, Kingdom of Saudi Arabia; 2 Department of Nutritional Sciences, School of Public Health, University of Michigan, Ann Arbor, Michigan, United States of America; 3 Department of Health Sciences, Clinical Nutrition Program, College of Health and Rehabilitation Sciences, Princess Nourah Bint Abdulrahman University, Riyadh, Kingdom of Saudi Arabia; Universidad de Extremadura Facultad de Enfermeria y Terapia Ocupacional, SPAIN

## Abstract

We investigated dietary patterns and their associations with sociodemographic and lifestyle factors in Saudi adults with type 2 diabetes mellitus (T2DM). A total of 297 participants (154 men and 143 women) with a mean age (standard deviation) of 54.0 (7.0) years were enrolled in the current study. Self-reported information on sociodemographic and lifestyle characteristics, eating behavior, and frequency of consumption was collected from all participants. Principal component analysis was used to determine dietary patterns. Multiple linear regressions were used to examine the associations between dietary patterns and sociodemographic and lifestyle factors. We identified five dietary patterns: “Vegetables and olive oil,” “Refined grains and sweets,” “Dairy products and legumes,” “Dates and beverages,” and “Fruit.” Age was inversely associated with the pattern “Refined grains and sweets” (β = -0.036, p <0.001). Females were more likely to have higher scores with the “Fruit” pattern (β = 0.410, p = 0.011). Smoking was associated with “Vegetables and olive oil” (β = 0.919, p = 0.001) and “Dates and beverages” (β = -0.947, p = 0.001) patterns. The level of physical activity was associated with “Dates and beverages” (β = 0.104, p = 0.048) and “Vegetables and olive oil” (β = -0.102, p = 0.048) patterns. The number of snacks consumed was associated with “Dates and beverages” pattern (β = -0.241, p = 0.005), and the frequency of meals eaten in fast food locations and restaurants per month was associated with “Vegetables and olive oil” pattern (β = -0.043, p = 0.034). Each dietary pattern was associated with different sociodemographic and lifestyle factors and eating behaviors. This study provides insights into the underlying nutritional habits of Saudi Arabian patients with T2DM. Future studies are necessary to assess these associations in representative samples.

## Introduction

According to the International Diabetes Federation in 2019, the age-adjusted prevalence of diabetes in the Middle East and North Africa region (MENA) was estimated at 12.2% (54.8 million people), which is the highest prevalence when compared with other regions [1]. Furthermore, diabetes accounted for 1.5 million deaths worldwide in 2019 [2]. Saudi Arabia (SA) has 4.3 million people with diabetes; this figure is the fourth highest among MENA countries. In 2019, the estimated age-adjusted prevalence of diabetes in SA was 15.8%, with a total medical cost of 5,012,600 USD. There were approximately 15,039 deaths attributed to diabetes, which represented 68.5% of deaths in people under 60 years of age. The projected prevalence of diabetes in 2030 and 2045 is 17.2% and 17.8%, respectively [3, 4]. SA has undergone socioeconomic changes in recent decades, which have resulted in changes in eating habits and lifestyle patterns [5]. Diet is a modifiable factor that can prevent or delay the incidence of type 2 diabetes mellitus (T2DM) or any chronic disease [2, 6]; Therefore, it is crucial to examine the relationship between diet and diabetes to deliver public health recommendations [6].

It has previously been shown that there is an association of single food and beverages with the risk of T2DM, such as whole grains [7, 8], coffee, dairy products [8, 9], red meat, processed meat [8, 10], sugary-sweetened beverages [8, 11, 12], vegetables [8], and fruits [8]. However, diet is a “multi-dimensional” exposure [13], where food contains multiple nutrients and bioactive components [14] and food intake is captured by meals and snacks patterns [15–17]. As a result, dietary pattern analysis has been proposed as an alternative to the traditional approaches [15]. Dietary patterns allow understanding food combinations and account for interactions between food and nutrients [13, 15–17]. Furthermore, this approach will allow researchers to examine the cumulative and holistic role of diet to prevent or control health outcomes [13, 15], and then provide easily understood health recommendations to the lay public [6, 15, 17, 18]. There are two forms of analysis for dietary patterns. The first is called the *a posteriori* approach, where statistical methods are applied to explore common dietary patterns and food combinations among the studied populations. Examples of these analytical approaches are factor, cluster analysis, and reduced-rank regression [19]. The second approach is called the *a priori* approach, which assesses the conformity of an individual’s diet with established dietary guidelines or recommendations [6, 13–16, 18, 20, 21].

To tailor public health messages or design dietary interventions for adults with T2DM, it is of great importance to begin with the fundamental step of identifying common dietary patterns. To our knowledge, data on the association between dietary patterns and T2DM in the Saudi population are limited. Consumption patterns are determined by socioeconomic characteristics, beliefs, cultural norms, the environment, geographic locations, and other factors [22]. Therefore, the objective of this cross-sectional study was to explore the dietary patterns among adults with T2DM in Riyadh, Saudi Arabia, and to examine their associations with sociodemographic and lifestyle factors. This research will help design effective diabetes prevention programs to maintain overall health and well-being in SA.

## Materials and methods

### Study design and population

A convenience sample of 297 adults diagnosed with T2DM, who were visiting King Abdulaziz University Hospital, Riyadh, SA were recruited to participate in this study. The data collection was conducted between June and December 2013 (Rajab [7^th^ month of the Hijri Calendar] 1434–Safar [2^nd^ month of the Hijri Calendar] 1435). Due to changes in eating patterns during the holy month of Ramadan fasting, the data collection was put on hold and resumed afterward. Pregnant or lactating women and subjects on any diet that restricted the consumption of any food groups (e.g., gluten-free diet, ketogenic diet) were excluded from the study. Written informed consent was obtained from all participants at the time of enrollment. The Institutional Review Board of the Medical College at King Saud University approved the study (IRB # 13/3757 /IRB, project number E-10-152).

The research staff administered a questionnaire to each participant. The questionnaire consisted of sociodemographic information (i.e., age, sex, level of education, marital status, and monthly income), lifestyle information (i.e., smoking status, physical activity, and sleeping duration), dietary behaviors, and health status information. A trained nurse in the clinic measured height and weight using an appropriate international standard scale (Digital Person Scale, ADAM Equipment Inc., USA). Body mass index (BMI) was calculated as weight / height squared (kg/m^2^).

### Dietary assessment

A trained nutritionist administered a validated food frequency questionnaire (FFQ) to collect habitual dietary information over the previous six months [23]. The frequency of consumption for each food item was captured with four possible responses: per day, per week, per month, or rarely/never [23]. Visual aids were provided to estimate portion size: food models (Fort Atkinson, WI, USA), household units (e.g., bowls, spoons, and cups), and pictures (Nelson, MAFF, UK). Nutrient composition was analyzed using a nutritional analysis program (Q-Builder V2.0, Tinuviel Software Ltd., UK).

### Covariates

Self-reported demographic characteristics were marital status (married, others—including single, widowed, and divorced), education measured as the highest completed level of education (secondary education or less, higher education, and more), occupation status (working either in the governmental or private sector, not working—includes unemployed and retired), and monthly income (< 10.000 Saudi riyals [SR], ≥ 10,000 SR). Lifestyle behaviors included smoking status (yes, no—includes former smokers), physical activity (no, 1–2 times/week, 3–4 times/week, ≥ 5 times/week), and sleeping hours per day (≤ 5 hrs., > 5–7 hrs., > 7 hrs.). Health indicators were the duration of diagnosis of T2DM (years) and complications of T2DM (no, yes, including retinopathy, neuropathy, nephropathy, cardiovascular diseases, hypertension, hyperlipidemia). Dietary behaviors were captured using the following variables: visiting a dietitian (no, yes), following a diabetic diet (no, yes), number of meals per day (1, 2, 3), number of snacks per day (1, 2, 3), skipped meals (no, sometimes, yes), most frequently skipped meals (breakfast, lunch, dinner), frequent meals eaten outside the home (breakfast, lunch, dinner), meals eaten outside the home per month (continuous), meals eaten at fast food locations and restaurants per month (continuous), and breakfast eaten per week (continuous).

### Statistical analysis

Dietary patterns were derived from the collected FFQ data. A factor analysis method was used to derive the food patterns. A total of 157 FFQ food items were consolidated into 36 food groups based on their nutritional compositions (S1 Table). We used principal component analysis to reduce the 36 food groups into factors and used orthogonal rotation via the varimax procedure to improve interpretation [24]. Of the generated factors, the first five factors were retained after careful examination of the eigenvalues, scree plot, percentage of the variance explained by each factor, and the interpretability of the identified patterns. A food group with a factor load of ≥ 0.30 was considered a key group to describe the pattern.

Mean ± (standard deviation) and frequency (percentage) were reported for continuous and categorical variables, respectively. Partial Pearson’s correlation coefficients were used to determine the relationship between each dietary pattern and nutrients, adjusting for total energy, age, and sex. Linear regression models were used to examine the relationship between each dietary pattern and demographic and lifestyle characteristics, adjusting for potential confounding factors. The SPSS statistical software package, version 22, was used for the analysis (IBM Corp., NY, USA), and a P-value < 0.05 was considered statistically significant.

## Results

Tables 1 and 2 summarize the sociodemographic and lifestyle factors of the study population stratified by sex. This analysis included 279 adults; females accounted for 48% of the sample. The mean (standard deviation) for age, BMI, and duration of diabetes was 53.74 (6.54) years, 31.17 (5.06) kg/m^2^, and 12.75 (7.97) years, respectively (Table 1). Less than 5% of the participants were smokers and 40% were physically inactive. The most common health conditions reported were dyslipidemia and hypertension. A dietitian saw approximately 70% of the subjects. Of the three main meals, breakfast was the most skipped meal and dinner was the meal most consumed away from home (Table 2).

**Table 1:** Sociodemographic characteristics of the study population stratified by sex.

	Total (*n =* 297)		Male (*n =* 154)		Female (*n =* 143)	
	Frequency	%	Frequency	%	Frequency	%
**Age** *	53.74	6.54	54.03	6.61	53.43	6.46
**Marital status**						
Married	257 ^a^	86.53	149 ^b^	96.75	108 ^c^	75.52
Others (includes single, widowed, and divorced)	36 ^a^	12.12	2 ^b^	1.30	34 ^c^	23.78
**Educational level**						
Completed high school or less	200	67.34	77	50	123	86.01
Higher education or more	97	32.66	77	50	20	13.99
**Occupation status**						
Working	145	48.82	119	77.27	26	18.18
Not working (unemployed and retired)	152	51.18	35	22.73	117	81.82
**Monthly income**						
< 10,000 SR	118	39.73	34	22.08	84	58.75
≥ 10,000 SR	179	60.27	120	77.92	59	41.26
**BMI (kg/m** ^**2**^ **)** *	31.17	5.06	30.13	4.53	32.28	5.37
**Duration of T2DM (years)** *	12.75 ^d^	7.97	12.28 ^e^	8.10	13.26	7.82
**Complications of T2DM**	261	87.88	147	95.45	137	95.80

* mean and standard deviation

^a^n = 293

^b^n = 151

^c^n = 142

^d^n = 296

^e^n = 153

**Table 2:** Lifestyle Characteristics of the study population stratified by sex.

	Total (*n =* 297)		Male (*n =* 154)		Female (*n =* 143)	
	Frequency	%	Frequency	%	Frequency	%
**Smoking status**	13	4.38	13	8.44	0	0
**Physical activity**						
No	120	40.4	46	29.9	74	51.7
1–2 times a week	76	25.6	42	27.3	34	23.8
3–4 times a week	46	15.5	33	21.4	13	9.1
≥ 5 times a week	55	18.5	33	21.4	22	15.4
**Sleeping hours per day**						
≤ 5 hours	39	13.13	13	8.44	26	18.18
> 5 - < 7 hours	84	28.28	47	30.52	37	25.87
> 7 hours	174	58.59	94	61.04	80	55.94
**Visited a dietitian**	203	68.35	107	69.48	96	67.13
**Followed a diabetic diet**	100	33.67	44	28.57	56	39.16
**No. of meals per day**						
One meal	16 ^a^	5.39	6 ^b^	3.90	10 ^c^	6.99
Two meals	82 ^a^	27.61	26 ^b^	16.88	56 ^c^	39.16
Three meals	177 ^a^	59.60	117 ^b^	75.97	60 ^c^	41.96
**No. of snacks per day**						
One snack	111 ^d^	37.41	61 ^e^	39.6	50 ^f^	35
Two snacks	113 ^d^	38	51 ^e^	33.1	62 ^f^	43.4
Three snacks	33 ^d^	11.1	20 ^e^	13.00	13 ^f^	9.1
**Skipped meals**						
No	91 ^g^	30.64	63 ^h^	40.91	28 ^i^	19.58
Sometimes	99 ^g^	33.33	60 ^h^	38.96	39 ^i^	27.27
Yes	78 ^g^	26.26	22 ^h^	14.29	56 ^i^	39.16
**Most frequently skipped meals**						
Breakfast	73	24.58	27	17.53	46	32.17
Lunch	42	14.14	15	9.74	27	18.88
Dinner	68	22.90	40	25.97	28	19.58
**Meals eaten outside the home**						
Breakfast	10 ^j^	3.37	8 ^k^	5.19	2 ^l^	1.40
Lunch	16 ^j^	5.39	11 ^k^	7.14	5 ^l^	3.50
Dinner	122 ^j^	41.08	72 ^k^	46.75	50 ^l^	34.97
**Meals eaten outside of the house per month** *	3.13	2.8	3.70	3.32	2.27	1.86
**Meals eaten at fast food locations and restaurants per month** *	3.6 ^m^	4.1	4.1 ^n^	4.7	2.7 ^o^	2.4
**Breakfast consumed per week** *	5.22	2.70	5.9	2.11	4.5	3.05

* mean and standard deviation

^a^n = 275

^b^n = 149

^c^n = 126

^d^n = 257

^e^n = 132

^f^n = 125

^g^n = 268

^h^n = 145

^i^n = 123

^j^n = 148

^k^n = 91

^l^n = 57

^m^n = 152

^n^n = 94

^o^n = 58

### Association between dietary patterns and diet composition

Table 3 shows the five identified dietary patterns which collectively explained 60% of the variance in dietary intake among the study population. The variance explained by each pattern ranged from 21% for the “Vegetables and olive oil” pattern to 7% for the “Fruit” pattern. The second pattern was labeled “Refined grains and sweets”, which consisted of refined grains, sweets, and desserts, and this pattern was negatively correlated with whole grains consumption. The “Dairy products and legumes” pattern reflected the intake of these two food groups. The “Dates and beverages” pattern consisted of coffee—Arabic coffee—tea, and dates. The last pattern was labeled “Fruit,” which captured the intake of whole fruits (Table 3).

**Table 3:** Factor loads for diet patterns^a^^,^^b^.

Food Groups	Dietary Patterns				
	Vegetables & olive oil	Refined grains & sweets	Dairy products & legumes	Dates & beverages	Fruit
Vegetables, others	0.828				
Vegetables, green leafy	0.764				
Olive oil	0.446				
Vegetables, dark yellow	0.359				
Tomatoes	0.311				
Grains, refined		0.898			
Grains, whole		-0.681			
Sweets and desserts		0.333			
Dairy products			0.928		
Legumes			0.312		
Tea				0.766	
Arabic coffee				0.635	
Dates				0.502	
Fruit					0.961
Variance explained (%)	20.98	12.24	11.1	8.29	7.41

^a^Values are factor loadings obtained from the analysis of the principal components

^b^Food groups included that have factor loadings ≥ 0.30 within a given dietary pattern

Table 4 presents the correlation between each dietary pattern and the nutrients composition. We found a positive correlation between caloric intake and each dietary pattern; therefore, we adjusted our models for total caloric intake in addition to age and sex. “Vegetable and olive oil” patterns were positively correlated with the intake of fiber, monounsaturated fat, minerals (i.e., sodium, calcium, iron), and vitamins (i.e., carotene, vitamin E, folate, and vitamin C), and negatively correlated with the intake of saturated fat, retinol, and vitamin B12. The “Refined grains and sweets” pattern was negatively correlated with the intake of fiber, minerals (i.e., calcium and iron), and vitamins (i.e., carotene and folate). The third dietary pattern, “Dairy products and legumes,” was positively correlated with a higher protein, cholesterol, sodium, calcium, iron, and vitamin B12 intake. The pattern “Dates and traditional beverages” was positively correlated with the intake of carbohydrates and polyunsaturated fat, and negatively with the intake of cholesterol, saturated fat, retinol, and vitamin B12. Lastly, the pattern of “Fruits” was positively correlated with the intake of carbohydrates, fiber, vitamin E, folate, and vitamin C but negatively correlated with that of protein, total fat, and monounsaturated fat (Table 4)

**Table 4:** Correlation between dietary pattern and diet composition^a^.

	Vegetables & olive oil		Refined grains & sweets		Dairy products & legumes		Dates & beverages		Fruit	
	R	P-value	r	P-value	R	P-value	r	P-value	r	P-value
Protein (g)	0.105	0.071	-0.061	0.294	0.182	**0.002**	-0.038	0.517	-0.120	**0.039**
Carbohydrate (g)	-0.025	0.669	0.004	0.943	-0.056	0.336	0.129	**0.027**	0.194	**0.001**
Fat (g)	-0.021	0.722	0.015	0.804	-0.005	0.926	-0.113	0.054	-0.154	**0.008**
Total fiber (g)	0.476	**<0.001**	-0.385	**<0.001**	0.110	0.060	0.096	0.100	0.298	**<0.001**
Cholesterol (mg)	-0.073	0.209	0.069	0.239	0.118	**0.043**	-0.163	**0.005**	-0.090	0.122
Saturated fat (g)	-0.147	**0.011**	0.010	0.863	0.025	0.672	-0.196	**0.001**	-0.112	0.055
Monounsaturated fat (g)	0.127	**0.029**	-0.016	0.781	-0.051	0.386	-0.050	0.395	-0.129	**0.027**
Polyunsaturated fat (g)	0.082	0.158	0.043	0.466	-0.029	0.617	0.128	**0.028**	-0.107	0.067
Sodium (mg)	0.161	**0.006**	-0.088	0.131	0.266	**<0.001**	-0.065	0.270	-0.072	0.219
Calcium (mg)	0.312	**<0.001**	-0.174	**0.003**	0.365	**<0.001**	-0.007	0.907	-0.031	0.592
Iron (mg)	0.244	**<0.001**	-0.182	**0.002**	0.160	**0.006**	0.031	0.594	0.095	0.103
Retinol (μg)	-0.121	**0.037**	0.097	0.098	0.072	0.216	-0.125	**0.032**	-0.004	0.946
Carotene (μg)	0.453	**<0.001**	-0.142	**0.015**	-0.029	0.623	0.047	0.419	-0.033	0.578
Vitamin E (mg)	0.430	**<0.001**	-0.037	0.522	-0.070	0.233	0.053	0.362	0.130	**0.025**
Vitamin B12 (μg)	-0.049	0.402	0.053	0.367	0.148	**0.011**	-0.151	**0.010**	0.042	0.478
Folate (μg)	0.563	**<0.001**	-0.317	**<0.001**	0.100	0.086	0.032	0.587	0.170	**0.004**
Vitamin C (mg)	0.132	**0.024**	-0.088	0.133	0.021	0.725	-0.049	0.407	0.295	**<0.001**

^a^Correlations were adjusted for age, sex, and total caloric intake

Gram = g; milligram = mg; microgram = μg

### Association between dietary patterns, sociodemographic, and lifestyle factors

Table 5 shows the cross-sectional between each dietary pattern and sociodemographic, and lifestyle factors. The “Vegetables and olive oil” pattern was positively associated with smoking status [β = 0.919, p = 0.001], but inversely related to the frequency of meals eaten at fast food and restaurants per month [β = -0.043, p = 0.034] and physical activity [β = -0.102, p = 0.048]. Age was inversely associated with the “Refined grains and sweets” pattern [β = -0.036, p <0.001]. “Dates and beverages” pattern was inversely associated with smoking status [β = -0.947, p = 0.001] and the number of snacks consumed [β = -0.241, p = 0.005], but it was positively associated with the level of physical activity [β = 0.104, p = 0.048]. The “Fruit” pattern was positively associated with females [β = 0.410, p = 0.011]. No associations were detected between the “Dairy products and legumes” pattern and any of the sociodemographic and lifestyle factors (Table 5).

**Table 5:** Association between sociodemographic and lifestyle characteristics and dietary pattern^a^.

	Vegetables & olive oil		Refined grains & sweets		Dairy products & legumes		Dates & beverages		Fruit	
	β	P-value	β	P-value	Β	P-value	β	P-value	β	P-value
Age	0.014	0.160	-0.036	**<0.001**	0.011	0.245	0.005	0.637	0.012	0.260
Female	0.034	0.826	-0.006	0.968	0.127	0.396	0.246	0.122	0.410	**0.011**
Marital Status	0.204	0.328	-0.141	0.511	-0.308	0.124	-0.030	0.888	-0.074	0.733
Educational level	-0.035	0.812	0.004	0.979	-0.247	0.084	-0.246	0.104	0.138	0.367
Monthly income	-0.073	0.630	-0.043	0.780	0.033	0.819	-0.080	0.603	0.093	0.551
Duration of T2DM	-0.007	0.423	-0.015	0.071	0.010	0.232	0.009	0.300	-0.001	0.945
Complications of T2DM	-0.021	0.912	0.123	0.531	-0.065	0.722	-0.010	0.958	0.017	0.931
Smoking status	0.919	**0.001**	0.400	0.174	-0.397	0.149	-0.947	**0.001**	-0.046	0.876
Sleeping hours	0.094	0.083	0.055	0.323	0.037	0.475	-0.021	0.696	-0.087	0.119
Physical activity per week	-0.102	**0.048**	0.024	0.649	0.018	0.721	0.104	**0.048**	-0.076	0.155
Body mass index	0.006	0.675	0.002	0.894	-0.025	0.056	-0.001	0.963	0.026	0.069
No. of meals per day	-0.136	0.250	-0.001	0.992	-0.110	0.331	0.031	0.794	-0.012	0.923
No. of snacks per day	-0.044	0.599	-0.007	0.938	0.016	0.844	-0.241	**0.005**	-0.089	0.307
Meals eaten outside of the house per month	0.067	0.059	0.030	0.404	0.049	0.148	-0.050	0.164	-0.010	0.792
Fast food and restaurants per month	-0.043	**0.034**	0.006	0.770	0.024	0.213	0.033	0.108	-0.004	0.835
Breakfast per week	0.015	0.593	-0.006	0.833	0.054	0.050	0.015	0.598	0.031	0.300

^a^All sociodemographic and lifestyle variables were run in one multivariate model

Type 2 diabetes mellitus = T2DM

## Discussion

To our knowledge, this is the first exploratory study conducted to identify common dietary patterns and examine their associations with sociodemographic and lifestyle factors, and eating behaviors among adults with T2DM in SA. Five dietary patterns emerged, explaining 60% of the overall variance in food intake. The “Vegetables and olive oil” pattern accounted for the highest variance, followed by the “Refined grains and sweets,” “Dairy products and legumes,” “Dates and beverages,” and “Fruit” patterns. The correlations between the nutritional compositions of the identified dietary patterns explained the labeling.

Diet is a cornerstone in the management of T2DM [25]. Substantial evidence from prospective and randomized studies highlights the vital role of healthy dietary patterns in the prevention and management of T2DM [20, 26]. The common characteristics of recommended dietary patterns comprise the consumption of fruit, vegetables, and other plant-based foods and the avoidance or moderate consumption of animal-based products [20, 26]. In our study, two of the five identified patterns encompassed these recommendations, namely the “Vegetables and olive oil” and “Fruit” patterns. Similar dietary patterns have emerged in other studies [19, 27]. Our data showed that the “Vegetables and olive oil” and “Fruit” patterns were positively associated with fiber, vitamin, and mineral intake, which is consistent with the high contents of these nutrients in fruit and vegetables [28]. Conversely, the high olive oil consumption in our study is inconsistent with the low olive oil consumption reported in a previous cross-sectional study involving Saudi male adults (N = 80) with and without cardiovascular diseases [29]. The difference between the two study populations may be a reason for this discrepancy. The importance of olive oil consumption might be more strongly emphasized in dietary recommendations for adults with T2DM, given that it has been associated with improved glucose homeostasis [30–32]. As 70% of our sample received nutrition education for T2DM management, they were more likely to receive emphasis on the benefits of consuming olive oil.

A substantial body of evidence suggests the protective role of whole grains in glycemic control and the prevention of T2DM [7, 33–36] as they are rich in fibers, vitamins, and minerals [33]. We found that the “Refined grains and sweets” pattern explained 12% of the variability in intake, and was inversely correlated with the composition of whole grains. This finding confirms the low compliance with the dietary recommendations, which has been previously reported in Riyadh [37–39], Medina [40], Taif [41], and Abha [42]. Low adherence to diabetes dietary recommendations has been highlighted in other studies on adults with T2DM in SA. Alhaiti et al. showed that only 36.9% of T2DM patients received advice to increase fruit and vegetable consumption, and less than 50% were instructed to consume complex carbohydrates, eat a high-fiber diet, or reduce sweet consumption [37]. Although Al Johani et al. reported higher proportions, they found that only 12% of the participants incorporated diet as a daily self-care behavior [40], they found that only 12% of the participants incorporated diet as a daily self-care behavior [40]. Adherence to pharmacological treatment scores higher than any lifestyle component among Saudis with T2DM [37–41]; therefore, our study supports the calls for healthcare providers to emphasize the role of lifestyle factors in the management of T2DM [37–42]. Furthermore, it is necessary to involve the patients themselves in the management plan to increase their awareness, as self-efficacy is positively associated with T2DM-related self-care behaviors [38, 39]. Another benefit can be derived from helping patients overcome hindrances related to eating styles and social and cultural norms, which have been suggested to interfere with adherence to self-care practices for T2DM among Saudis [37, 38, 40].

Sociodemographic factors were associated with some dietary patterns identified in this study. We found an inverse association between age and the “Refined grains and sweets” pattern and a positive association between the female sex and the “Fruits” pattern. Our age results align with the findings reported by studies in Brazil [43, 44], New Zeeland [45], Lebanon [46], France [19], and Norway [47]. It has been suggested that because older age is associated with chronic diseases and more frequent visits to health care facilities, the chances of receiving recommendations about health and nutrition increase. Exposure to health information may result in better diet quality and habits [44]. Previous studies have reported higher fruit consumption among women than among men [48, 49]. Regarding eating habits, participants with high scores in the “Dates and beverages” pattern consumed fewer snacks. We think people in SA were less likely to consider dates and Arabic coffee consumption as snacks. Lower consumption of fast food was commonly reported by our participants with high scores in the “Vegetables and olive oil” pattern.

Regarding lifestyle factors, smoking was positively associated with the “Vegetables and olive oil” pattern and inversely associated with the “Dates and beverages” pattern. These results are not consistent with previous reports of negative associations between smoking and healthy dietary patterns [19, 46], and higher diet quality among former smokers [43, 44]. However, associations with smoking have been shown to be selective. Marques-Vidal et al. reported that, despite an inverse association between smoking and “Fruit and vegetables,” there were no positive associations with the unhealthy “Meat and fries,” and “Fatty and sugary” patterns [19]. Associations between smoking and dietary patterns have usually been found in groups with specific sociodemographic and lifestyle characteristics. A representative sample of New Zealand adults showed that a “traditional” pattern was positively associated with smoking and food insecurity, and inversely associated with education [45]. Among Norwegian adults, a “Fish/Traditional” pattern was positively associated with age, being male, smoking, higher education, a small household size, and overweight/obesity, while a “Sweets and bakery” pattern was directly associated with older age, being female, and large household size and negatively associated with higher education level, high income, smoking, and overweight/obesity [47]. In our study, none of the women reported active smoking status, and as our results show, there are differences in dietary consumption between sexes. Our findings may not be comparable with those of other studies that included smokers of both sexes. In terms of physical activity, we found a borderline positive association between physical activity and “Dates and beverages” patterns and a negative association with “’Vegetables and olive oil” pattern. Two previous studies reported that physical activity was positively associated with healthy patterns and inversely associated with unhealthy patterns [46, 47]. Both studies evaluated physical activity using validated questionnaires, as opposed to our self-report method [46, 47]. Further studies on the holistic relationship between sociodemographic and lifestyle characteristics and dietary patterns are warranted.

Our study has some limitations that merit discussion. The study’s cross-sectional design did not allow us to establish a causal relationship between dietary patterns and sociodemographic and lifestyle characteristics. Also, this study may have been underpowered to detect associations due to the relatively small sample size, which prevented us from conducting sex-stratified analyses. In addition, we acknowledge the identified dietary patterns and their associations with sociodemographic and lifestyle characteristics among a convenience sample cannot be generalized to the target population. However, we designed our study as an exploratory step and a hypothesis-generating tool for future well-designed prospective and intervention studies that can contribute to improving dietary intake among adults with T2DM. Another limitation is that, due to the use of principal component analysis, we made multiple subjective decisions. Examples include constructing our 36 food groups out from the FFQ items and selecting and labeling the dietary factors. However, subjectivity is an inherent characteristic of this statistical method and applies to all studies that use it [24].

Despite our efforts to reduce the selection bias and the nonresponse rate and ensure a consistent understanding, the presence of the research staff is inevitably a cause for concern about social desirability bias, which may have resulted in misreporting intake (i.e., over-reporting the consumption of foods that are consistent with a healthy diabetic dietary plan and under-reporting incompatible foods). Additionally, the prior nutritional counseling provided to our population may have influenced the reported food consumption in a way that reflected greater adherence to dietetic recommendations than the patients’ actual dietary patterns (i.e., learning effect). Moreover, we acknowledge that our data may not precisely reflect current eating patterns as they were collected several years prior. Future studies may identify changes in eating patterns over time. Nevertheless, our study makes a useful contribution to the literature, as it is the first to examine dietary patterns among Saudi adults diagnosed with T2DM and could be used as a reference for comparisons. Finally, as is the case with any observational study, we cannot rule out the possibility of residual confounders due to unmeasured or crudely measured covariates.

Despite these limitations, this is to our knowledge, one of the few explanatory studies on the relationship between dietary patterns and sociodemographic and lifestyle characteristics among Saudi adults. We derived dietary patterns using the principal component analysis, which is commonly used in epidemiological studies, and allowed us to examine the variability of eating habits [15]. Our patterns explained 60% of the intake variance, leaving only 40% of the variance uncaptured. We used validated semi-quantitative FFQs to determine the usual intake, including the consumption of local foods [23], which allowed us to investigate seasonal food consumption variability.

Our study reveals common dietary patterns in a convenience sample of Saudi adults with T2DM. Our explanatory analysis highlights the need to place more emphasis on incorporating lifestyle education (e.g., diet and physical activity) into T2DM management. More studies are needed to contribute to improving adherence to dietary recommendations among adults with T2DM. As controlling for the learning effect on reported food consumption is crucial, future studies could stratify their analyses by exposure to nutrition education to identify any differences in reported food consumption between patients receiving nutrition education and patients not receiving such education. Finally, representative population-based studies on consumption patterns in different regions of SA are warranted.

## Supporting information

S1 Table Food groupings used for dietary pattern analysis. (DOCX)
