# Supplementary material for: Dietary patterns among Saudis with type 2 diabetes mellitus in Riyadh: A cross-sectional study
Source: PLoS One. 2022 May 5;17(5):e0267977. doi: 10.1371/journal.pone.0267977 (PMC9070904; doi:10.1371/journal.pone.0267977)
Supplement: S1 Table — (DOCX) [file pone.0267977.s001.docx]

## **S1 Table:** Food groupings used for dietary pattern analysis.

| **No** | **Food group** | **Food items** |
| --- | --- | --- |
| 1 | Butter | - Margarine - Butter - Cream - Liquid cheese |
| 2 | Cream soup | - Creamed soup chicken or mushroom. |
| 3 | Coffee | - Arabic coffee |
| 4 | Cold breakfast cereal | - Breakfast cereals - Special cereals breakfast |
| 5 | Condiments | - Ketchup - Mayonnaise - Pickles |
| 6 | Dairy products | - Labneh* - White cheese - Processed cheese - Milk - Laban - Yogurt - Yogurt with cucumber |
| 7 | Date | - Date |
| 8 | Drinks | - Soft drink - Diet soft drink - Energy drinks - Fruit drink (canned or packed juice) - Nescafe or other coffees - Coffee Mate |
| 9 | Eggs | - Eggs (All types). |
| 10 | Fish | - Fried fish - Grilled fish - Tuna (Canned in oil or water) - Shrimp |
| 11 | Fruit | - Melon - Watermelon - Apple - Orange - Mandarin - Banana - Grapes - Pears - Apricot - Peach - Strawberry |
| 12 | Fruit, others | - Fruit salad - Dried fruit - Fresh fruit juice |
| 13 | Grains, refined | - White bread (Pitta Bread, Mafrood*) - Roll white (Samoli*) - White toast - Tamees* - Fateer* - Shaborah* white (Rusk) - Fatayer* (labneh*, Spinach, Thyme) - Croissants - Rice plain boiled - Rice (kabsah*, biryani*, bokhari*, mandy*) - Pasta plain boiled - Pasta with tomato sauce |
| 14 | Grain, refined, others | - Pasta with meat - Pasta Béchamel/ Lasagna - Sambosa* (cheese, meat) - Mutbaq* - koshai - Couscous |
| 15 | Grains, whole | - Brown bread (Pitta Bread, mafrood*) - Roll brown (Samoli*) - Brown toast - Shaborah* brown (Rusk) - Bran corn flakes - Whole bran cereals - Oat soup - Jareesh* soup (Crushed wheat) |
| 16 | Legumes | - Balilah* (Chickpeas) - Hummus* (Chickpeas) - Foul* (Fava beans) - Lentils - Falafel - Lentils soup |
| 17 | Nuts | - Nuts - Peanut butter |
| 18 | Olive oil | - Olive oil - Olive |
| 19 | Meat | - Beef meats (All types) - Lamb - Camel - Variety meat & liver - Kebbah* - Kamounia* (beef and liver stew prepared with cumin) |
| 20 | Poultry | - Chicken meats (All types) |
| 21 | Pizza | - Pizza |
| 22 | Potatoes | - Potatoes (peeled and boiled) - Potatoes with Béchamel |
| 23 | Potatoes, fried | - Fried potatoes |
| 24 | Processed meats | - Mortadella - Hot dog (Frankfurters/Sausage) |
| 25 | Salad dressing, and others | - Salad dressing - Tehinah* |
| 26 | Snacks | - Tea biscuits or digestive - Salty biscuits - Popcorn - Crackers (Chips) |
| 27 | Sweets and desserts | - Honey - jam - Muhallebi* (rice pudding with milk) or cream caramel - Arabic desserts - Maamoul* dates - Cinnamon roll - Cake - Gateau cake - Doughnut - Chocolate (Any type) - Ice-cream (Any type) - Candy (Gum, Lollypop) - Biscuit with Choco or cream |
| 28 | Tea | - Tea |
| 29 | Tomatoes | - Tomatoes |
| 30 | Traditional food | - Gareesh* - Haress* - Qursan* - Mataziz* - Mashghotha* - Areka* - Saleeq* - Manto* - Yagmush* - Aseedah borr* - Maasoob* - Mohala* - Henini* - Aseeda tamer* |
| 31 | Vegetables, cruciferous | - Cauliflower cooked - Cabbage with carrot - Cabbage |
| 32 | Vegetables, dark-yellow | - Carrot - Pumpkin cooked - Squash cooked |
| 33 | Vegetables, green leafy | - Lettuce - Spinach cooked - tabouleh * - [Mulukhiyah](https://www.google.com/search?q=%E2%80%A2%09Mulukhiyah&spell=1&sa=X&ved=2ahUKEwiX2PDFqcD2AhX4gf0HHYkaBeEQkeECKAB6BAgCEDE) * cooked - Watercress |
| 34 | Vegetables, others | - Onion - Celery (pascal large outer stalk) - Cucumber - Fattoush* - Green salad - Corn cooked - Mixed vegetable cooked - Okra cooked - Peas and carrots cooked - Green beans cooked - Vegetable oven cooked - Metablbazngan* (eggplant) - Vegetable soup |
| 35 | Vegetables, mixed | - Stuffed vegetables - Grape leaves stuffed - Vegetable with Béchamel |
| 36 | Oil, other unsaturated | - Vegetable oil |

* Arabic dishes
